# Risk of hypoglycaemia in type 2 diabetes patients under different insulin regimens: a primary care database analysis

**DOI:** 10.3205/000205

**Published:** 2015-01-12

**Authors:** Karel Kostev, Franz W. Dippel, Wolfgang Rathmann

**Affiliations:** 1 IMS HEALTH, Frankfurt, Germany; 2 Department of Internal Medicine, Neurology und Dermatology, University of Leipzig, Germany; 3 Institute of Biometrics and Epidemiology, German Diabetes Center, Duesseldorf, Germany

**Keywords:** insulin therapy, type 2 diabetes, hypoglycaemia, risk factors, primary care

## Abstract

**Aims:** To compare rates and predictors of documented hypoglycaemia in type 2 diabetes patients treated with either basal insulin supported oral therapy (BOT), conventional therapy (CT) or supplementary insulin therapy (SIT) in primary care.

**Methods:** Data from 10,842 anonymous patients (mean age ± SD: 54 ± 8 yrs) on BOT, 2,407 subjects (56 ± 7 yrs) on CT, and 7,480 patients (52 ± 10 yrs) using SIT from 1,198 primary care practices were retrospectively analyzed (Disease Analyzer, Germany: 01/2005–07/2013). Stepwise logistic regression (≥1 documented hypoglycaemia: ICD code) was used to evaluate risk factors of hypoglycemia.

**Results:** The unadjusted rates (95% CI) per 100 patient-years of documented hypoglycaemia were 1.01 (0.80–1.20) (BOT), 1.68 (1.10–2.30) (CT), and 1.61 (1.30–1.90) (SIT), respectively. The odds of having ≥1 hypoglycemia was increased for CT (OR; 95% CI: 1.71; 1.13–2.58) and SIT (1.55; 1.15–2.08) (reference: BOT). Previous hypoglycemia (OR: 11.24; 6.71–18.85), duration of insulin treatment (days) (1.06; 1.05–1.07), history of transient ischemic attack (TIA)/stroke (1.91; 1.04–3.50), and former salicylate prescriptions (1.44; 1.06–1.98) also showed an increased odds of having hypoglycemia. Higher age was associated with a slightly lower odds ratio (per year: 0.98; 0.97–0.99).

**Conclusions:** Insulin naïve type 2 diabetes patients in primary care, initiated with CT and SIT have an increased risk of hypoglycaemia compared to BOT, which is in line with previous randomized controlled trials. As hypoglycaemic events are associated with an increased mortality risk, this real-world finding is of clinical relevance.

## Zusammenfassung

**Zielsetzung:** Das Ziel dieser Studie war es die Raten und Prädiktoren dokumentierter Hypoglykämien bei Patienten mit Typ-2-Diabetes (T2D) mit einer basalunterstützen oralen Therapie (BOT), einer konventionellen Insulintherapie (CT) oder einer supplementären Insulintherapie (SIT) in primärärztlichen Praxen zu untersuchen.

**Methodik:** Aus 1.198 hausärztlichen Praxen wurden Datensätze von 10.842 anonymisierten Patienten (mittleres Alter 54 ± 8 Jahre) mit BOT, 2.407 Patienten (56 ± 7 Jahre) mit CT und 7.480 Patienten (52 ± 10 Jahre) mit SIT retrospektiv untersucht (Disease Analyzer, Deutschland: 01/2005–07/2013). Um die Risikofaktoren für Hypoglykämie zu untersuchen wurde schrittweise logistische Regression angewendet.

**Ergebnisse:** Die unadjustierten Raten (95%-KI) pro 100 Patientenjahre für die erfassten Hypoglykämien betrugen 1,01 (0,80–1,20) (BOT), 1,68 (1,10–2,30) (CT) und 1,61 (1,30–1,90) (SIT). Die Chance für Hypoglykämie war erhöht für CT (Odds ratio; OR; 95% KI: 1,71; 1,13–2,58) und SIT (1,55; 1,15–2,08) (Referenz: BOT). Vorangegangene Hypoglykämien (OR: 11,24; 6,71–18,85), Dauer der Insulintherapie (in Tagen) (1,06; 1,05–1,07), frühere transitorische ischämische Attacke (TIA)/Apoplex (1,91; 1,04–3,50) und Verschreibungen von Salicylaten (1,44; 1,06–1,98) erhöhen ebenfalls die Chance einer Hypoglykämie. Hingegen zeigt höheres Alter ein verringertes Odds Ratio für Hypoglykämien (pro Jahr: 0,98; 0,97–0,99).

**Schlussfolgerung:** T2D-Patienten in der Primärversorgung, die mit CT und SIT behandelt werden, haben im Vergleich zu BOT ein erhöhtes Hypoglykämie-Risiko, was mit früheren Studienergebnissen übereinstimmt. Da Hypoglykämien mit einer erhöhten Mortalitätsrate assoziiert sind, haben diese Real-World Daten eine hohe klinische Relevanz.

## Introduction

Hypoglycemia is a common side effect of insulin therapy in type 2 diabetes, and has a negative impact on mortality, morbidity and quality of life [1]. There is evidence from several randomized controlled clinical trials (RCTs) that the rates of severe and symptomatic hypoglycemia are different among various insulin treatment regimens, favoring long-acting insulin compared to premixed or short acting insulin use in type 2 diabetes [2], [3], [4]. However, these studies were conducted in subjects that may not be comparable to those in real-world primary care practices, where the majority of type 2 diabetes patients are treated. There is insufficient evidence outside clinical trials on the risk of hypoglycemic events in insulin-treated type 2 diabetes patients [5], [6].

Thus, the aim of the current analysis was to investigate the rates and predictors of documented hypoglycemia in patients with type 2 diabetes on various insulin treatment regimens in general and internal medicine practices.

## Patients and methods

The Disease Analyzer database (IMS HEALTH) assembles drug prescriptions, diagnoses, and basic medical and demographic data directly obtained from the practice computer system of general practitioners [7]. Diagnoses (ICD-10), prescriptions (Anatomical Therapeutic Chemical (ATC) Classification System) and the validity of reported data were monitored by IMS based on a number of quality criteria (e.g. completeness of documentation, linkage of diagnoses and prescriptions). In connection with data/figures used terms, such as “patient, doctor, medical practice, prescriber or pharmacy”, do not designate any personal data but exclusively anonymous information (in accordance with § 3 Abs. 6 “Bundesdatenschutzgesetz” – German Federal Data Protection Act)

The analyzed database period was January 2005 to July 2013 (1,198 practices). For the assessment of diabetes duration and comorbidity the whole patient history in the practice was considered (mean: 3 years). All insulin naïve patients (age 18–65 years) with type 2 diabetes (ICD-10 E11 and/or corresponding history of oral treatment) starting their first insulin treatment during the observational period (at least one prescription of a defined insulin) were eligible for the analysis. Patients were followed up until they either changed their insulin preparation or type of insulin (treatment regimen). First, the kind of insulin treatment regimen (basal supported oral therapy, BOT; conventional therapy with premixed insulin, CT; supplementary (short-acting prandial) insulin therapy, SIT) was assessed. Then, patients with hypoglycaemia were identified by specific ICD-10 codes (E16.0, E16.1, E16.2) among the three insulin treatment groups. The codes include drug-induced hypoglycemia (E16.0), other (E16.1: hyperinsulinism, functional nonhyperinsulinemic hypoglycemia) and unspecified hypoglycemia (E16.2).

Potential predictors of hypoglycemia considered in the present analysis were age, sex, diabetes duration (>5 years), duration of insulin treatment (days) in the practices, private health insurance, history of transient ischemic attack (TIA) or stroke and co-medication with oral antidiabetic drugs. In addition, the Charlson comorbidity index was used as general marker of comorbidity. The Charlson score is a weighted index that accounts for the number and severity of comorbidities in administrative database studies [8]. The conditions included in the Charlson index cover a wide range of comorbidities (macrovascular diseases, dementia, pulmonary diseases, gastrointestinal, liver and renal diseases, diabetes, tumors and AIDS). Finally, the potential association of salicylate prescriptions (ATC: B1C1, N2B2) with hypoglycemia was assessed. The main indications for salicylates in the practices were coronary heart disease (35%), cerebrovascular disease (15%), and peripheral vascular disease (9%).

Descriptive statistics (means, standard deviations, median and interquartile range, proportions) are given for the above mentioned variables. The proportion of patients with at least one documented hypoglycemia and the event rates per 100 patient-years were calculated. Logistic regression models were fitted with hypoglycemia (≥1 during 12 months) as dependent variable and the potential predictors. Variance inflation factor (VIF) was used to identify multicollinearity for the multivariate regression model. No VIF greater than 10 was accepted. A final model was fitted using stepwise logistic regression. Two sided tests were used and a p-value of <0.05 was considered as statistically significant. All analyses were carried out using SAS 9.3. (SAS Institute, Cary, USA). The analysis was carried out following established national [9] and international good practice recommendations of secondary data analysis [10].

## Results

The clinical characteristics of the type 2 diabetes patients stratified by kind of insulin treatment regimen (BOT, CT, SIT) are shown in Table 1 (Tab. 1). Patients with CT were slightly older than those with BOT, and patients with SIT were younger than the BOT group (both p<0.05).

The average period of insulin treatment and the proportion with long-standing diabetes (>5 years) were significantly increased in BOT users compared to CT or SIT (both p<0.05). As expected, co-medication with oral antidiabetic agents was more often observed in the BOT group (p<0.05). Salicylates were slightly more often prescribed among CT than in BOT, and were less often used in SIT (p<0.05). History of TIA or stroke were also more often found in CT than in BOT users and were less frequently diagnosed in the SIT group (p<0.05). The Charlson comorbidity score showed no significant differences between the three insulin groups. Finally, a pervious history of documented hypoglycaemia was found in about 1% of patients first treated with BOT and SIT and was less often reported in patients initiated on CT (0.5%; p<0.05 vs. BOT). 

The prevalence of patients (95% CI) with at least one documented hypoglycaemia during the follow-up was 0.8% (95% CI: 0.6–1.0%) in BOT, 1.3% (0.9–1.8%) in CT, and 1.3% (1.1–1.6%) in SIT (p<0.001 CT vs. BOT; p<0.001 SIT vs. BOT). Overall, 87 (BOT), 32 (CT), and 100 (SIT) hypoglycaemic events were documented during the study period, respectively. The unadjusted rates (95% CI) per 100 patient-years of documented hypoglycaemia were 1.01 (0.80–1.20) (BOT), 1.68 (1.10–2.30) (CT), and 1.61 (1.30–1.90) (SIT), respectively.

The results of the logistic regression analysis including all variables are shown in Table 2 (Tab. 2). The odds of having at least one documented hypoglycaemia was significantly increased for CT and SIT compared to BOT. Longer duration of insulin treatment, previous hypoglycaemia using oral drugs, and history of TIA/stroke were also independently related to an increased odds. A borderline statistical significance was found for the Charlson comorbidity score (Table 2 (Tab. 2)). Previous salicylate prescriptions were significantly associated with an increased odds of having hypoglycemia. Finally, higher age was related to a lower odds of hypoglycemic events, e.g. a 10-year increase of age was associated with a 20% lower risk of hypoglycemia in the insulin-treated type 2 diabetes patients. The variance inflation factors for all variables were low (<1.5) indicating no multicollinearity.

Then, a final multivariate model was fitted using stepwise logistic regression (Table 3 (Tab. 3)). History of previous hypoglycemia was the strongest predictor. Both CT and SIT (reference: BOT) were related to an increased odds of having hypoglycemia. Furthermore, longer insulin treatment and previous TIA/stroke were related to increased odds of having hypoglycemia. Prescription use of salicylates also showed a significantly increased odds ratio. Finally, age was related to a lower odds of hypoglycemia in multivariable analysis. 

## Discussion

This real-world study shows that the odds of having a documented hypoglycaemia was higher in type 2 diabetes patients who initiated a conventional therapy (CT) with premixed insulin or were started on short acting insulin (SIT) than in those who began a basal insulin supported oral therapy (BOT). There was also evidence for an increased odds of having hypoglycaemia for patients with a history of TIA or stroke. In addition, previous salicylate prescriptions were independently related to a higher odds of having hypoglycaemia. 

These results add to the limited body of evidence from previous observational studies [5], [6]. Administrative data from self-insured employers in the US were previously used to analyse the risk of severe hypoglycaemic events (requiring inpatient or emergency department care) related to different insulin types [5]. In Cox regression analyses adjusting for potential confounders, premixed insulin (hazard ratio, HR: 2.12) and rapid acting insulin (HR: 2.75) showed significantly higher risks of severe hypoglycaemia compared with a long-acting insulin analogue [5]. Furthermore, in a propensity score analysis of a multinational survey in 12 countries (CREDIT study: Europe, Canada, Japan), the relative risk both of overall and nocturnal hypoglycaemia was lower with basal insulin compared with premixed insulin [6]. In addition, there were fewer nocturnal hypoglycaemic events among those treated with basal (only) compared to patients with basal and mealtime insulin [6]. 

An important difference between the present and previous studies is the ascertainment of hypoglycaemia. Most likely, the hypoglycaemic diagnoses recorded in the Disease Analyzer primary care database mainly reflect symptomatic and severe events. It has been estimated that only 15% of hypoglycaemic episodes in patients with type 2 diabetes are reported to the doctor [1]. Our results are in line with a previous smaller survey from Germany on self-reported severe hypoglycaemic events (SHE) during the last year [11]. The mean (SD) annual number of SHE was 0.2 (0.6) in BOT, 0.5 (2.0) in SIT, and 0.5 (3.2) in other (mixed) insulin therapies [11].

The present study also indicated several comorbidities and co-medication that were related to increased odds of experiencing hypoglycaemia. History of TIA or stroke was independently related to an increased odds of having hypoglycaemia. In line with this finding, the prevalence of patients with stroke was higher among those with severe hypoglycaemic events (6.8%) compared with insulin-treated type 2 diabetes patients without severe events (3.3%) in the previous study from the US [5]. The strongest association in the present study was found for history of previous hypoglycaemia, which is also confirming previous findings [12]. 

Salicylate prescription use was also related to an increased hypoglycaemia risk. Salicylates may induce hypoglycaemia due to increased insulin secretion and sensitivity [13]. In concomitant use with sulfonylureas, salicylates may also displace the oral antidiabetic agent from its protein-binding sites and may inhibit its renal excretion [13]. Thus, the present study indicates that the potential for salicylate-induced hypoglycaemia should be considered in insulin-treated type 2 diabetes. Finally, higher age was related to a lower odds of hypoglycaemia (2% per year). Further studies are necessary to evaluate the underlying reasons for this association. It is conceivable that this relationship may reflect a higher HbA1c target in older patients in order to avoid severe hypoglycemia.

## Study limitations

Retrospective primary care database analyses are in general limited by the validity and completeness of data. As an example, no valid information on diabetes type and prescribed daily insulin doses were available in the database. Also assessment of comorbidity relied on ICD-10 codes by primary care physicians only. Data on socioeconomic status (e.g. education, income) and lifestyle-related risk factors (e.g. smoking, alcohol, physical activity) were also lacking. Finally, HbA1c and body mass index values were only available for a subgroup and were not considered. 

## Conclusion

In conclusion, type 2 diabetes patients in primary care with CT and SIT have an increased risk of hypoglycaemia compared to BOT. As hypoglycaemic events are associated with an increased mortality risk, this real-world finding should be considered by primary care physicians when selecting among the various alternative insulin regimens for their patients.

## Notes

### Competing interests

The study was supported by an unrestricted grant from Sanofi-Aventis, Berlin, Germany. The authors declare that they have no competing interests.

## Figures and Tables

**Table 1:**
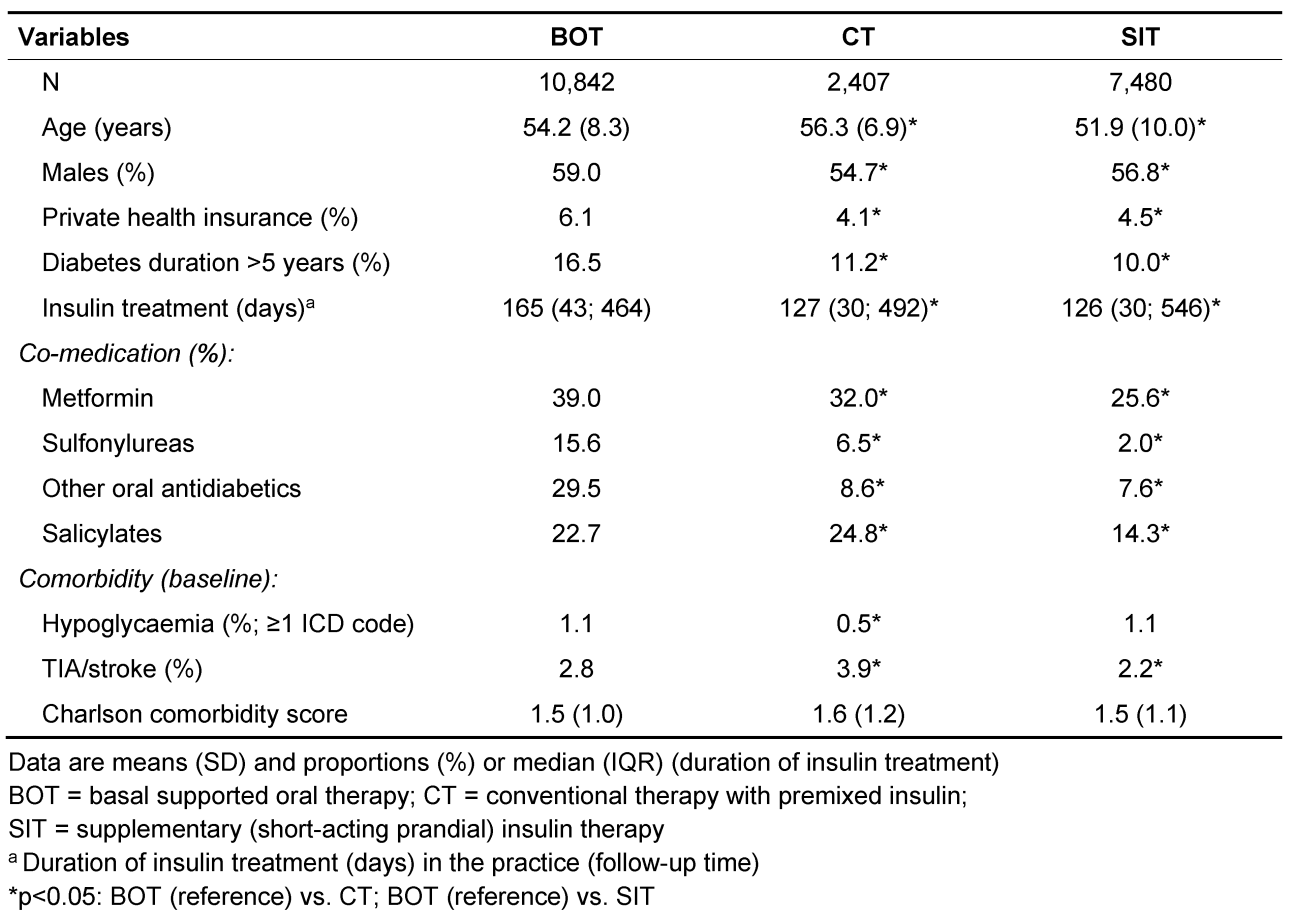
Baseline characteristics of insulin-treated type 2 diabetes patients: IMS HEALTH Disease Analyser database, Germany

**Table 2:**
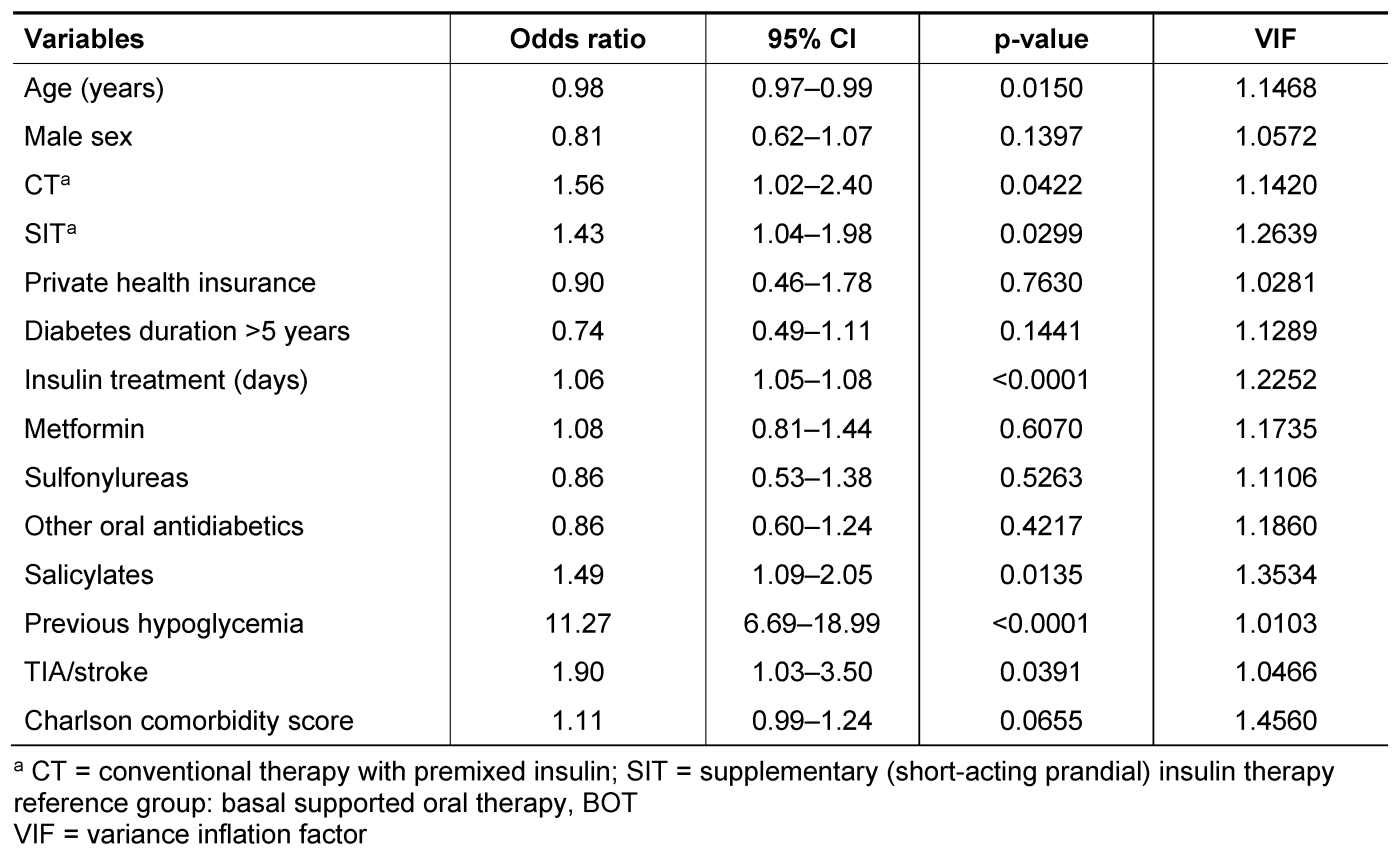
Association of potential risk factors with documented hypoglycaemia in insulin-treated type 2 diabetes in primary care: multivariate logistic regression analyses

**Table 3:**
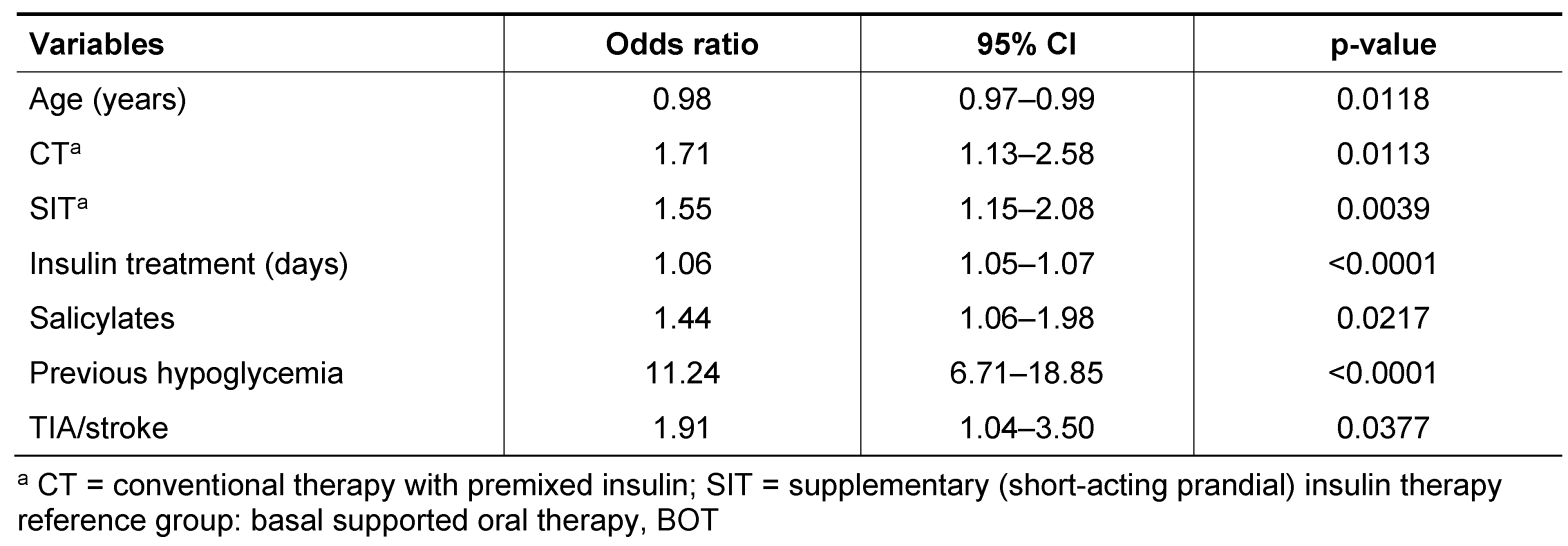
Association of potential risk factors with documented hypoglycaemia in insulin-treated type 2 diabetes in primary care: multivariate logistic regression analyses (stepwise selection)
